# Crystal structure of an apremilast ethanol hemisolvate hemihydrate solvatomorph

**DOI:** 10.1107/S2056989017006661

**Published:** 2017-05-09

**Authors:** Yun-Deng Wu, Xiao-Hong Liu, Jian Xu, Si-Han Zhang, Kun Shen, Ling Sun, Yong-Mei He, Yan Ma, Ai-Hua Zhang

**Affiliations:** a SpringPharma Tech Co. Ltd, Weidi Road 9, 210046 Nanjing, People’s Republic of China; b JiangSu Center for Safety Evaluation of Drugs, Majia Street 26, 210009 Nanjing, People’s Republic of China

**Keywords:** apremilast, PDE4, psoriatic arthritis, crystal structure, hydrogen bonding, solvatomorph

## Abstract

The title compound, C_22_H_24_N_2_O_7_S·0.5C_2_H_5_OH·0.5H_2_O {systematic name: (*S*)-4-acetamido-2-[1-(3-eth­oxy-4-meth­oxy­phen­yl)-2-(methyl­sulfon­yl)eth­yl]iso­indo­line-1,3-dione ethanol hemisolvate hemihydrate}, is a novel solvatomorph of apremilast (AP), which is an inhibitor of phosphodiesterase 4 (PDE4) and is indicated for the treatment of adult patients with active psoriatic arthritis. The asymmetric unit contains one mol­ecule of AP and disordered mol­ecules of ethanol and water, both with half occupancy. The dihedral angle between the planes of the phenyl ring and the iso­indole ring is 67.9 (2)°. Extensive intra- and inter­molecular hydrogen bonds help to stabilize the mol­ecular conformation and sustain the crystal packing.

## Abstract 2

The title compound represents another solvatomorph of apremilast, containing half of an ethanol and half of a water solvent mol­ecule per formula unit

## Chemical context   

Analogues of thalidomide have been reported to possibly enhance tumor necrosis factor alpha (TNFα) inhibitory activity (Corral *et al.*, 1996 ▸; Muller *et al.*, 1996 ▸) and phospho­diesterase type 4 (PD4) inhibition (Muller *et al.*, 1998 ▸), hence showing potential for the treatment of inflammatory diseases (de Brito *et al.*, 1997 ▸). Among these substances are phen­ethyl­sulfones substituted in the α position to the phenyl group with a 1-oxoisoindoline or 1,3-dioxoisoindoline group that can reduce the levels of TNFα in a mammal. Typical embodiments are (*S*)-2-[1-(3-eth­oxy-4-meth­oxy­phen­yl)-2-(meth­yl­sulfon­yl)eth­yl]-4-acetamido­isoindoline-1,3-dione] with the generic name apremilast (AP), which is an inhibitor of phosphodiesterase 4 (PDE4) and is indicated for the treatment of adult patients with active psoriatic arthritis (Gottlieb *et al.*, 2008 ▸; Man *et al.*, 2009 ▸; Duplantier *et al.*, 1996 ▸). In our previous studies, we reported three solvatomorphs of AP with ethyl acetate, toluene and di­chloro­methane, respectively (Wu *et al.*, 2017 ▸). However, these three solvates exhibit toxicity, in particular the solvates of toluene and di­chloro­methane, which clearly limits the possibility of these compounds being developed into drugs. In a continuation of our work, a novel solvatomorph of AP with ethanol and water solvents in the molar ratio 1:0.5:0.5 was prepared and its crystal structure determined. This solvatomorph of AP appears to be suitable for development into a powerful drug, showing much lower toxicity than the solvatomorphs of ethyl acetate, toluene and di­chloro­methane.
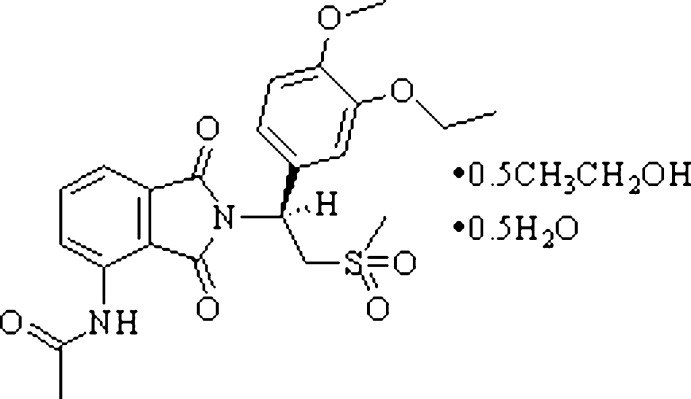


## Structural commentary   

**Figure 1:**
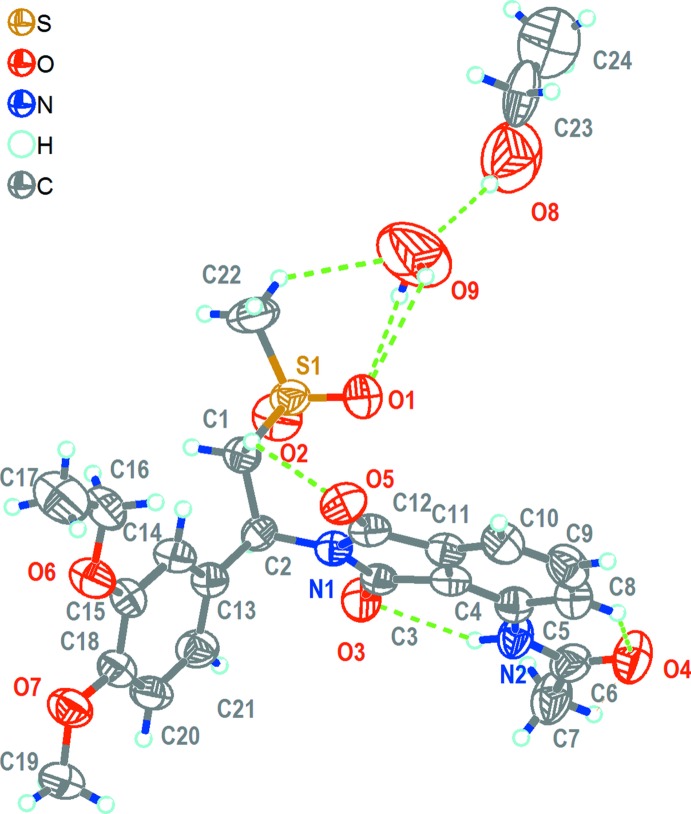
The structures of the mol­ecular components in (I). Displacement ellipsoids are drawn at the 50% probability level. Hydrogen bonds are shown as dashed lines.

**Figure 2:**
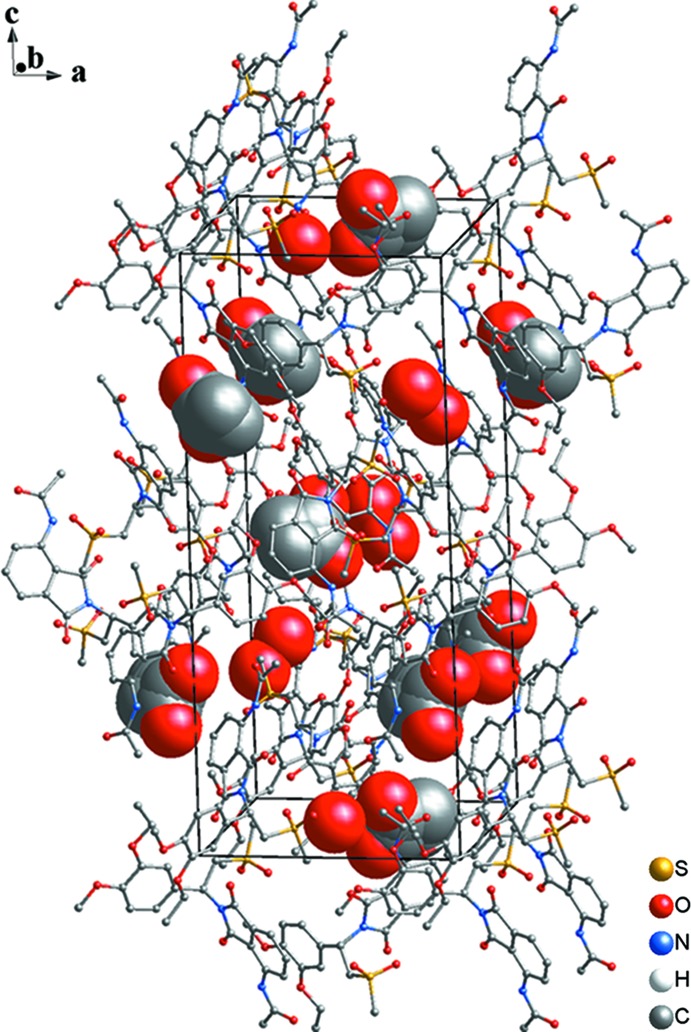
The unit cell of (I), with the solvent mol­ecules shown in space-filling mode. [See Table 1 ▸ for symmetry codes.]

The title solvatomorph (I) crystallizes in the same space group (*P*4_1_2_1_2) as the other three structurally characterized solvatomorphs of ethyl acetate, toluene and di­chloro­methane (Wu *et al.*, 2017 ▸). The structures of the mol­ecular components of (I) are shown in Fig. 1 ▸. The asymmetric unit comprises one mol­ecule of AP and one solvent mol­ecule each of ethanol and water, both being disordered about a twofold rotation axis (occupancy for both solvent mol­ecules = 0.5). A space-filling drawing of the structure is given in Fig. 2 ▸, emphasizing the positions of the solvent mol­ecules in the crystal structure. The bond lengths and angles in the AP mol­ecule are in normal ranges and very similar to those in the previous three solvatomorphs (Wu *et al.*, 2017 ▸). The same applies to the dihedral angle between the phenyl (C13–C20) and iso­indole (C3–C5/C8–C12/N1) rings, which is 67.9 (2)° in the title structure. The conformation of the AP mol­ecule is stabilized by several intra­molecular hydrogen bonds of types N—H⋯O and C—H⋯O (Table 1 ▸).

## Supra­molecular features   

**Figure 3:**
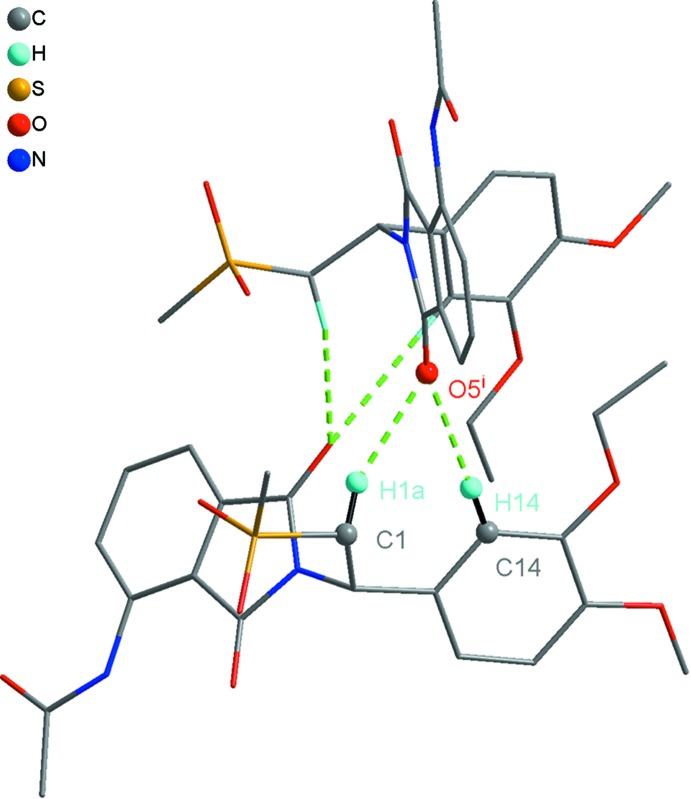
(AP)_2_ dimers with an 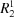
(7) motif formed by C—H⋯O hydrogen bonds. [See Table 1 ▸ for symmetry code.]

**Figure 4:**
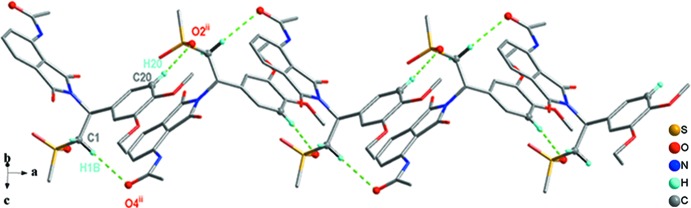
C1—H1*B*⋯O4^ii^ and C20—H20⋯O2^ii^ hydrogen bonds incorporating 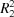
(18) motifs expand the structure parallel to the *a*-axis direction. [See Table 1 ▸ for symmetry codes.]

An extensive network of inter­molecular hydrogen-bonding inter­actions exists in the crystal structure (Figs. 1 ▸, 2 ▸–4; Table 1 ▸). The water mol­ecule (O9) is hydrogen-bonded to the AP mol­ecule by C22—H22*C*⋯O9 and O9—H9*B*⋯O1 inter­actions and likewise is bonded by an O8—H8*A*⋯O9 inter­action to the ethanol solvent mol­ecule. As well as these hydrogen bonds involving the solvent mol­ecules, there are inter­actions between AP mol­ecules. Two AP mol­ecules are arranged into a dimer with an 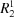
(7) motif (Fig. 3 ▸) by C—H⋯O hydrogen bonds, and a zipper-like chain including 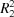
(18) motifs (Fig. 4 ▸) is formed parallel to the *a* axis by additional C—H··O hydrogen bonds.

## Synthesis and crystallization   

AP was prepared according to a literature protocol (Muller *et al.*, 2006 ▸, 2008*a* ▸,*b* ▸). A 100 ml round-bottomed flask equipped with a magnetic stirring bar was charged with a solution of (*S*)-1-(3-eth­oxy-4-meth­oxy­phen­yl)-2-methyl­sulfonyl­ethan­amine *N*-acetyl leucine salt (5.0 g, 11.2 mmol, 1.0 eq) and 3-acetamido­phthalic anhydride (2.42 g, 11.8 mmol, 1.05 eq) to which glacial acetic acid (50 ml) was added. The mixture was refluxed for 16 h and then cooled to room temperature. The solvent was removed *in vacuo*, and the residue was dissolved in ethyl acetate. The resulting solution was washed with water (2 × 50 ml), saturated aqueous sodium bicarbonate (2 × 50 ml), brine (2 × 50 ml), and dried over anhydrous sodium sulfate. The solvents were evaporated *in vacuo*, and the obtained AP recrystallized from an ethanol/acetone mixture (2:1, *v*/*v*). Single crystals of (I) were obtained by slow evaporation of an AP-saturated solution from an *N*,*N*-di­methyl­formamide/ethanol/water mixture (1:10:2, *v*/*v*/*v*), at room temperature over 90 days.

## Refinement   

Crystal data, data collection and structure refinement details are summarized in Table 2 ▸. Hydrogen atoms bound to nitro­gen or carbon atoms were placed in calculated positions (N—H = 0.87, C—H = 0.93–0.98 Å) and constrained to ride on their carrier atoms [*U*_iso_(H) = 1.2*U*_eq_(C,N) or 1.5*U*_eq_(C_meth­yl_)]. Hydrogen atoms bound to oxygen atoms were deduced from difference-Fourier maps and their positions relative to donor and possible acceptor atoms. They were refined with *U*_iso_(H) = 1.5*U*_eq_(O). The solvent ethanol and water mol­ecules are disordered about a twofold rotation axis and were refined with an occupancy of 0.5. To get reasonable shape and displacement parameters for both mol­ecules, they were treated with DFIX, RIGU and ISOR restraints in *SHELXL2014* (Sheldrick, 2015*b* ▸).

## Supplementary Material

Crystal structure: contains datablock(s) I. DOI: 10.1107/S2056989017006661/wm5386sup1.cif

Structure factors: contains datablock(s) I. DOI: 10.1107/S2056989017006661/wm5386Isup2.hkl

CCDC reference: 1504229

Additional supporting information:  crystallographic information; 3D view; checkCIF report

## Figures and Tables

**Table 1:** Hydrogen-bond geometry (Å, °)

*D*—H⋯*A*	*D*—H	H⋯*A*	*D*⋯*A*	*D*—H⋯*A*
N2—H2⋯O3	0.86	2.31	2.986 (7)	136
O8—H8*A*⋯O9	0.92	2.30	3.20 (3)	166
O9—H9*B*⋯O1	0.89	2.54	2.994 (17)	112
C8—H8⋯O4	0.93	2.30	2.894 (9)	121
C1—H1*A*⋯O5	0.97	2.45	3.068 (8)	121
C1—H1*A*⋯O5^i^	0.97	2.32	3.172 (8)	147
C1—H1*B*⋯O4^ii^	0.97	2.56	3.524 (9)	172
C14—H14⋯O5^i^	0.93	2.49	3.415 (8)	178
C19—H19*C*⋯O4^iii^	0.96	2.61	3.567 (10)	173
C20—H20⋯O2^ii^	0.93	2.46	3.370 (9)	166
C22—H22*C*⋯O3^iv^	0.96	2.44	3.088 (9)	124
C22—H22*C*⋯O9	0.96	2.61	3.36 (2)	136

Symmetry codes: (i) 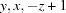; (ii) 
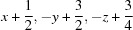
; (iii) 
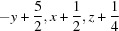
; (iv) 
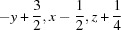
.

**Table 2:** Experimental details

Crystal data	
Chemical formula	C_22_H_24_N_2_O_7_S·0.5C_2_H_6_O·0.5H_2_O
*M*_r_	492.53
Crystal system, space group	Tetragonal, *P*4_1_2_1_2
Temperature (K)	298
*a*, *c* (Å)	12.9905 (18), 29.942 (6)
*V* (Å^3^)	5052.8 (17)
*Z*	8
Radiation type	Mo *K*α
μ (mm^−1^)	0.18
Crystal size (mm)	0.3 × 0.3 × 0.2

Data collection	
Diffractometer	Bruker P4
No. of measured, independent and observed [*I* > 2σ(*I*)] reflections	9575, 4390, 2811
*R*_int_	0.079
(sin θ/λ)_max_ (Å^−1^)	0.592

Refinement	
*R*[*F* ^2^ > 2σ(*F* ^2^)], *wR*(*F* ^2^), *S*	0.066, 0.183, 1.05
No. of reflections	4390
No. of parameters	329
No. of restraints	30
H-atom treatment	H-atom parameters constrained
Δρ_max_, Δρ_min_ (e Å^−3^)	0.62, −0.30
Absolute structure	Flack *x* determined using 833 quotients [(*I* ^+^)−(*I* ^−^)]/[(*I* ^+^)+(*I* ^−^)] (Parsons *et al.*, 2013 ▸)
Absolute structure parameter	0.15 (8)

Computer programs: *APEX2* and *SAINT* (Bruker, 2009 ▸), *SHELXT* (Sheldrick, 2015*a* ▸), *SHELXL2014* (Sheldrick, 2015*b* ▸) and *OLEX2* (Dolomanov *et al.*, 2009 ▸).

## supplementary crystallographic information

### Crystal data

**Table:**

C_22_H_24_N_2_O_7_S·0.5C_2_H_6_O·0.5H_2_O	*D*_x_ = 1.295 Mg m^−^^3^
*M**_r_* = 492.53	Mo *K*α radiation, λ = 0.71073 Å
Tetragonal, *P*4_1_2_1_2	Cell parameters from 2016 reflections
*a* = 12.9905 (18) Å	θ = 2–20°
*c* = 29.942 (6) Å	µ = 0.18 mm^−^^1^
*V* = 5052.8 (17) Å^3^	*T* = 298 K
*Z* = 8	Block, colourless
*F*(000) = 2080	0.3 × 0.3 × 0.2 mm

### Data collection

**Table:**

Bruker P4 diffractometer	θ_max_ = 24.9°, θ_min_ = 1.7°
ω scans	*h* = 0→15
9575 measured reflections	*k* = 0→15
4390 independent reflections	*l* = −35→35
2811 reflections with *I* > 2σ(*I*)	1 standard reflections every 60 reflections
*R*_int_ = 0.079	intensity decay: 1%

### Refinement

**Table:**

Refinement on *F*^2^	Hydrogen site location: mixed
Least-squares matrix: full	H-atom parameters constrained
*R*[*F*^2^ > 2σ(*F*^2^)] = 0.066	*w* = 1/[σ^2^(*F*_o_^2^) + (0.0992*P*)^2^] where *P* = (*F*_o_^2^ + 2*F*_c_^2^)/3
*wR*(*F*^2^) = 0.183	(Δ/σ)_max_ < 0.001
*S* = 1.05	Δρ_max_ = 0.62 e Å^−^^3^
4390 reflections	Δρ_min_ = −0.30 e Å^−^^3^
329 parameters	Absolute structure: Flack *x* determined using 833 quotients [(*I*^+^)-(*I*^-^)]/[(*I*^+^)+(*I*^-^)] (Parsons *et al.*, 2013)
30 restraints	Absolute structure parameter: 0.15 (8)

### Special details

**Table:**

Geometry. All esds (except the esd in the dihedral angle between two l.s. planes) are estimated using the full covariance matrix. The cell esds are taken into account individually in the estimation of esds in distances, angles and torsion angles; correlations between esds in cell parameters are only used when they are defined by crystal symmetry. An approximate (isotropic) treatment of cell esds is used for estimating esds involving l.s. planes.

### Fractional atomic coordinates and isotropic or equivalent isotropic displacement parameters (Å^2^)

**Table:**

	*x*	*y*	*z*	*U*_iso_*/*U*_eq_	Occ. (<1)
S1	0.89768 (15)	0.65667 (14)	0.46183 (6)	0.0537 (5)	
O3	0.9782 (4)	0.7422 (4)	0.33313 (15)	0.0565 (12)	
O6	1.2492 (4)	1.0317 (4)	0.51740 (15)	0.0650 (15)	
O5	0.8948 (4)	0.9600 (4)	0.44709 (16)	0.0618 (14)	
O1	0.8059 (4)	0.7045 (4)	0.44502 (17)	0.0690 (14)	
O2	0.9386 (4)	0.5729 (4)	0.43657 (18)	0.0728 (16)	
O4	0.6990 (5)	0.8281 (4)	0.21258 (18)	0.0774 (16)	
O7	1.3846 (4)	1.0504 (4)	0.45344 (16)	0.0660 (14)	
N1	0.9578 (4)	0.8403 (4)	0.39684 (16)	0.0425 (12)	
N2	0.8221 (4)	0.8054 (5)	0.26522 (17)	0.0544 (15)	
H2	0.8764	0.7699	0.2710	0.065*	
C3	0.9329 (5)	0.8089 (5)	0.3537 (2)	0.0418 (14)	
C2	1.0379 (5)	0.7928 (5)	0.4242 (2)	0.0441 (15)	
H2A	1.0637	0.7333	0.4075	0.053*	
C12	0.8912 (5)	0.9175 (5)	0.4107 (2)	0.0487 (16)	
C11	0.8173 (5)	0.9376 (5)	0.3739 (2)	0.0452 (15)	
C13	1.1290 (5)	0.8638 (5)	0.4319 (2)	0.0455 (16)	
C14	1.1440 (5)	0.9163 (5)	0.4720 (2)	0.0512 (17)	
H14	1.0952	0.9100	0.4945	0.061*	
C4	0.8437 (5)	0.8713 (5)	0.3392 (2)	0.0451 (16)	
C8	0.7059 (5)	0.9390 (5)	0.2964 (3)	0.0572 (18)	
H8	0.6670	0.9408	0.2703	0.069*	
C5	0.7893 (5)	0.8709 (5)	0.2996 (2)	0.0477 (16)	
C1	0.9941 (5)	0.7515 (5)	0.4684 (2)	0.0467 (15)	
H1A	0.9657	0.8086	0.4853	0.056*	
H1B	1.0501	0.7225	0.4858	0.056*	
C20	1.2874 (5)	0.9370 (6)	0.4048 (2)	0.0575 (18)	
H20	1.3353	0.9435	0.3819	0.069*	
C6	0.7810 (7)	0.7888 (6)	0.2243 (2)	0.0599 (19)	
C18	1.3020 (5)	0.9885 (5)	0.4443 (3)	0.0533 (18)	
C15	1.2291 (5)	0.9771 (5)	0.4791 (2)	0.0504 (17)	
C21	1.2011 (6)	0.8752 (5)	0.3988 (2)	0.0571 (19)	
H21	1.1921	0.8409	0.3718	0.069*	
C10	0.7368 (5)	1.0042 (6)	0.3713 (3)	0.0585 (18)	
H10	0.7199	1.0481	0.3947	0.070*	
C16	1.1741 (5)	1.0248 (6)	0.5523 (2)	0.065 (2)	
H16A	1.1668	0.9539	0.5619	0.078*	
H16B	1.1078	1.0488	0.5417	0.078*	
C9	0.6809 (6)	1.0032 (6)	0.3313 (3)	0.067 (2)	
H9	0.6249	1.0473	0.3282	0.081*	
C7	0.8411 (6)	0.7188 (7)	0.1951 (2)	0.076 (2)	
H7A	0.9067	0.7050	0.2086	0.114*	
H7B	0.8513	0.7508	0.1665	0.114*	
H7C	0.8043	0.6553	0.1912	0.114*	
C22	0.8740 (7)	0.6177 (7)	0.5166 (2)	0.083 (3)	
H22A	0.8596	0.6769	0.5347	0.124*	
H22B	0.9334	0.5827	0.5281	0.124*	
H22C	0.8159	0.5720	0.5171	0.124*	
C19	1.4549 (6)	1.0668 (8)	0.4174 (3)	0.086 (3)	
H19A	1.4204	1.1022	0.3936	0.128*	
H19B	1.4798	1.0017	0.4068	0.128*	
H19C	1.5118	1.1076	0.4277	0.128*	
C17	1.2096 (8)	1.0901 (9)	0.5904 (3)	0.104 (4)	
H17A	1.2163	1.1602	0.5807	0.157*	
H17B	1.2750	1.0656	0.6009	0.157*	
H17C	1.1602	1.0866	0.6142	0.157*	
O8	0.4584 (16)	0.3998 (17)	0.4571 (7)	0.177 (8)	0.5
H8A	0.5022	0.4544	0.4613	0.265*	0.5
C23	0.3896 (19)	0.3736 (17)	0.4889 (8)	0.108 (7)	0.5
H23A	0.3470	0.4341	0.4933	0.130*	0.5
H23B	0.4291	0.3653	0.5161	0.130*	0.5
C24	0.319 (3)	0.287 (3)	0.4878 (12)	0.164 (13)	0.5
H24A	0.3262	0.2506	0.4601	0.247*	0.5
H24B	0.2492	0.3117	0.4904	0.247*	0.5
H24C	0.3334	0.2412	0.5122	0.247*	0.5
O9	0.6400 (13)	0.5606 (12)	0.4753 (9)	0.192 (11)	0.5
H9A	0.6367	0.6301	0.4706	0.289*	0.5
H9B	0.7011	0.5451	0.4635	0.289*	0.5

### Atomic displacement parameters (Å^2^)

**Table:**

	*U*^11^	*U*^22^	*U*^33^	*U*^12^	*U*^13^	*U*^23^
S1	0.0592 (11)	0.0558 (11)	0.0461 (9)	−0.0131 (9)	−0.0010 (8)	0.0016 (8)
O3	0.057 (3)	0.067 (3)	0.045 (3)	0.013 (2)	0.002 (2)	−0.015 (2)
O6	0.060 (3)	0.082 (4)	0.053 (3)	−0.023 (3)	0.007 (2)	−0.021 (3)
O5	0.081 (3)	0.062 (3)	0.043 (3)	0.003 (3)	0.006 (2)	−0.014 (2)
O1	0.058 (3)	0.083 (4)	0.066 (3)	−0.008 (3)	−0.010 (3)	0.005 (3)
O2	0.085 (4)	0.058 (3)	0.076 (4)	−0.012 (3)	0.002 (3)	−0.017 (3)
O4	0.079 (4)	0.092 (4)	0.062 (3)	0.015 (3)	−0.021 (3)	−0.006 (3)
O7	0.054 (3)	0.080 (4)	0.064 (3)	−0.019 (3)	0.007 (3)	−0.004 (3)
N1	0.049 (3)	0.043 (3)	0.036 (3)	0.000 (3)	0.004 (2)	−0.007 (2)
N2	0.057 (4)	0.064 (4)	0.043 (3)	0.015 (3)	−0.006 (3)	−0.008 (3)
C3	0.042 (3)	0.046 (4)	0.037 (3)	−0.003 (3)	0.009 (3)	−0.002 (3)
C2	0.049 (4)	0.048 (4)	0.035 (3)	0.000 (3)	0.000 (3)	−0.005 (3)
C12	0.059 (4)	0.045 (4)	0.041 (4)	−0.010 (3)	0.008 (3)	−0.003 (3)
C11	0.048 (4)	0.043 (4)	0.044 (4)	−0.002 (3)	0.008 (3)	−0.006 (3)
C13	0.047 (4)	0.045 (4)	0.044 (4)	0.000 (3)	0.003 (3)	0.000 (3)
C14	0.051 (4)	0.052 (4)	0.051 (4)	−0.008 (3)	0.013 (3)	−0.004 (3)
C4	0.051 (4)	0.042 (4)	0.042 (4)	−0.006 (3)	0.011 (3)	−0.001 (3)
C8	0.054 (4)	0.054 (4)	0.063 (5)	0.003 (4)	−0.005 (4)	−0.003 (4)
C5	0.050 (4)	0.046 (4)	0.048 (4)	−0.001 (3)	0.003 (3)	0.001 (3)
C1	0.049 (4)	0.050 (4)	0.040 (3)	−0.007 (3)	−0.001 (3)	−0.004 (3)
C20	0.051 (4)	0.069 (5)	0.053 (4)	−0.007 (4)	0.013 (4)	0.006 (4)
C6	0.070 (5)	0.068 (5)	0.042 (4)	−0.008 (4)	−0.001 (4)	0.002 (4)
C18	0.046 (4)	0.055 (4)	0.059 (4)	−0.005 (4)	0.002 (3)	0.003 (3)
C15	0.041 (4)	0.055 (4)	0.055 (4)	−0.003 (3)	−0.001 (3)	−0.003 (3)
C21	0.063 (5)	0.065 (5)	0.043 (4)	−0.006 (4)	0.005 (3)	−0.006 (3)
C10	0.059 (5)	0.053 (4)	0.063 (5)	0.010 (4)	0.009 (4)	−0.010 (4)
C16	0.044 (4)	0.090 (6)	0.062 (5)	−0.006 (4)	0.004 (3)	−0.021 (4)
C9	0.053 (5)	0.059 (5)	0.090 (6)	0.011 (4)	0.007 (4)	−0.001 (4)
C7	0.079 (5)	0.098 (6)	0.051 (4)	0.024 (5)	−0.009 (4)	−0.017 (4)
C22	0.102 (7)	0.096 (7)	0.050 (5)	−0.029 (5)	0.010 (4)	0.017 (4)
C19	0.062 (5)	0.123 (8)	0.072 (6)	−0.027 (5)	0.004 (4)	0.023 (5)
C17	0.086 (7)	0.148 (9)	0.079 (7)	−0.024 (7)	0.014 (5)	−0.052 (6)
O8	0.161 (12)	0.190 (14)	0.180 (14)	0.097 (10)	−0.023 (11)	0.023 (12)
C23	0.110 (11)	0.086 (10)	0.129 (13)	0.061 (8)	−0.053 (9)	0.002 (10)
C24	0.179 (19)	0.150 (17)	0.16 (2)	0.014 (15)	−0.043 (16)	−0.010 (15)
O9	0.126 (14)	0.125 (14)	0.33 (3)	−0.030 (11)	0.032 (16)	0.084 (16)

### Geometric parameters (Å, º)

**Table:**

S1—O1	1.436 (5)	C20—H20	0.9300
S1—O2	1.428 (6)	C20—C18	1.372 (10)
S1—C1	1.768 (6)	C20—C21	1.390 (10)
S1—C22	1.744 (7)	C6—C7	1.485 (10)
O3—C3	1.214 (7)	C18—C15	1.417 (10)
O6—C15	1.373 (8)	C21—H21	0.9300
O6—C16	1.433 (8)	C10—H10	0.9300
O5—C12	1.221 (7)	C10—C9	1.400 (11)
O4—C6	1.232 (9)	C16—H16A	0.9700
O7—C18	1.368 (8)	C16—H16B	0.9700
O7—C19	1.430 (9)	C16—C17	1.495 (11)
N1—C3	1.394 (8)	C9—H9	0.9300
N1—C2	1.461 (8)	C7—H7A	0.9601
N1—C12	1.388 (8)	C7—H7B	0.9599
N2—H2	0.8600	C7—H7C	0.9602
N2—C5	1.401 (8)	C22—H22A	0.9600
N2—C6	1.353 (9)	C22—H22B	0.9600
C3—C4	1.478 (9)	C22—H22C	0.9600
C2—H2A	0.9800	C19—H19A	0.9600
C2—C13	1.518 (9)	C19—H19B	0.9600
C2—C1	1.539 (8)	C19—H19C	0.9600
C12—C11	1.485 (9)	C17—H17A	0.9600
C11—C4	1.392 (8)	C17—H17B	0.9600
C11—C10	1.360 (9)	C17—H17C	0.9600
C13—C14	1.394 (9)	O8—H8A	0.9182
C13—C21	1.372 (9)	O8—C23	1.35 (2)
C14—H14	0.9300	C23—H23A	0.9700
C14—C15	1.375 (9)	C23—H23B	0.9700
C4—C5	1.381 (9)	C23—C24	1.46 (2)
C8—H8	0.9300	C24—H24A	0.9600
C8—C5	1.401 (9)	C24—H24B	0.9600
C8—C9	1.377 (10)	C24—H24C	0.9600
C1—H1A	0.9700	O9—H9A	0.9150
C1—H1B	0.9700	O9—H9B	0.8918
			
O1—S1—C1	109.0 (3)	C20—C18—C15	119.4 (6)
O1—S1—C22	108.0 (4)	O6—C15—C14	125.3 (6)
O2—S1—O1	117.0 (3)	O6—C15—C18	115.7 (6)
O2—S1—C1	109.1 (3)	C14—C15—C18	118.9 (6)
O2—S1—C22	110.0 (4)	C13—C21—C20	121.3 (6)
C22—S1—C1	102.8 (4)	C13—C21—H21	119.3
C15—O6—C16	116.6 (5)	C20—C21—H21	119.3
C18—O7—C19	115.9 (6)	C11—C10—H10	121.9
C3—N1—C2	124.2 (5)	C11—C10—C9	116.3 (6)
C12—N1—C3	110.2 (5)	C9—C10—H10	121.9
C12—N1—C2	125.5 (5)	O6—C16—H16A	110.1
C5—N2—H2	115.3	O6—C16—H16B	110.1
C6—N2—H2	114.8	O6—C16—C17	108.2 (6)
C6—N2—C5	129.9 (6)	H16A—C16—H16B	108.4
O3—C3—N1	124.4 (6)	C17—C16—H16A	110.1
O3—C3—C4	128.5 (6)	C17—C16—H16B	110.1
N1—C3—C4	107.0 (5)	C8—C9—C10	122.2 (7)
N1—C2—H2A	106.9	C8—C9—H9	118.9
N1—C2—C13	112.7 (5)	C10—C9—H9	118.9
N1—C2—C1	111.5 (5)	C6—C7—H7A	109.4
C13—C2—H2A	106.9	C6—C7—H7B	109.4
C13—C2—C1	111.6 (5)	C6—C7—H7C	109.6
C1—C2—H2A	106.9	H7A—C7—H7B	109.5
O5—C12—N1	124.8 (6)	H7A—C7—H7C	109.5
O5—C12—C11	127.4 (6)	H7B—C7—H7C	109.4
N1—C12—C11	107.9 (5)	S1—C22—H22A	109.5
C4—C11—C12	106.6 (6)	S1—C22—H22B	109.5
C10—C11—C12	130.7 (6)	S1—C22—H22C	109.5
C10—C11—C4	122.7 (6)	H22A—C22—H22B	109.5
C14—C13—C2	122.5 (5)	H22A—C22—H22C	109.5
C21—C13—C2	119.2 (6)	H22B—C22—H22C	109.5
C21—C13—C14	118.3 (6)	O7—C19—H19A	109.5
C13—C14—H14	119.1	O7—C19—H19B	109.5
C15—C14—C13	121.8 (6)	O7—C19—H19C	109.5
C15—C14—H14	119.1	H19A—C19—H19B	109.5
C11—C4—C3	108.3 (6)	H19A—C19—H19C	109.5
C5—C4—C3	130.6 (6)	H19B—C19—H19C	109.5
C5—C4—C11	121.1 (6)	C16—C17—H17A	109.5
C5—C8—H8	119.6	C16—C17—H17B	109.5
C9—C8—H8	119.6	C16—C17—H17C	109.5
C9—C8—C5	120.8 (7)	H17A—C17—H17B	109.5
N2—C5—C8	124.6 (6)	H17A—C17—H17C	109.5
C4—C5—N2	118.5 (6)	H17B—C17—H17C	109.5
C4—C5—C8	116.9 (6)	C23—O8—H8A	120.7
S1—C1—H1A	108.7	O8—C23—H23A	105.6
S1—C1—H1B	108.7	O8—C23—H23B	105.6
C2—C1—S1	114.1 (4)	O8—C23—C24	127 (3)
C2—C1—H1A	108.7	H23A—C23—H23B	106.1
C2—C1—H1B	108.7	C24—C23—H23A	105.6
H1A—C1—H1B	107.6	C24—C23—H23B	105.6
C18—C20—H20	119.8	C23—C24—H24A	109.5
C18—C20—C21	120.3 (6)	C23—C24—H24B	109.5
C21—C20—H20	119.8	C23—C24—H24C	109.5
O4—C6—N2	122.3 (7)	H24A—C24—H24B	109.5
O4—C6—C7	122.7 (7)	H24A—C24—H24C	109.5
N2—C6—C7	115.1 (7)	H24B—C24—H24C	109.5
O7—C18—C20	124.7 (6)	H9A—O9—H9B	101.7
O7—C18—C15	116.0 (6)		
			
O3—C3—C4—C11	179.6 (6)	C12—C11—C10—C9	−179.1 (7)
O3—C3—C4—C5	−0.2 (11)	C11—C4—C5—N2	177.4 (6)
O5—C12—C11—C4	179.6 (6)	C11—C4—C5—C8	−1.0 (10)
O5—C12—C11—C10	−1.3 (12)	C11—C10—C9—C8	−0.4 (11)
O1—S1—C1—C2	−71.1 (6)	C13—C2—C1—S1	−173.0 (4)
O2—S1—C1—C2	57.7 (6)	C13—C14—C15—O6	179.5 (7)
O7—C18—C15—O6	1.6 (9)	C13—C14—C15—C18	1.6 (10)
O7—C18—C15—C14	179.7 (6)	C14—C13—C21—C20	0.2 (10)
N1—C3—C4—C11	−0.2 (7)	C4—C11—C10—C9	−0.2 (10)
N1—C3—C4—C5	180.0 (6)	C5—N2—C6—O4	−5.0 (12)
N1—C2—C13—C14	102.5 (7)	C5—N2—C6—C7	175.8 (7)
N1—C2—C13—C21	−79.2 (7)	C5—C8—C9—C10	0.3 (12)
N1—C2—C1—S1	60.1 (6)	C1—C2—C13—C14	−23.8 (9)
N1—C12—C11—C4	−0.3 (7)	C1—C2—C13—C21	154.5 (6)
N1—C12—C11—C10	178.8 (7)	C20—C18—C15—O6	−179.6 (6)
C3—N1—C2—C13	111.8 (6)	C20—C18—C15—C14	−1.5 (10)
C3—N1—C2—C1	−121.8 (6)	C6—N2—C5—C4	177.9 (7)
C3—N1—C12—O5	−179.8 (6)	C6—N2—C5—C8	−3.9 (12)
C3—N1—C12—C11	0.1 (7)	C18—C20—C21—C13	−0.1 (11)
C3—C4—C5—N2	−2.9 (11)	C15—O6—C16—C17	−179.4 (7)
C3—C4—C5—C8	178.7 (6)	C21—C13—C14—C15	−1.0 (10)
C2—N1—C3—O3	−3.8 (9)	C21—C20—C18—O7	179.5 (7)
C2—N1—C3—C4	176.1 (5)	C21—C20—C18—C15	0.8 (11)
C2—N1—C12—O5	4.3 (10)	C10—C11—C4—C3	−178.9 (6)
C2—N1—C12—C11	−175.8 (5)	C10—C11—C4—C5	0.9 (10)
C2—C13—C14—C15	177.3 (6)	C16—O6—C15—C14	−0.5 (10)
C2—C13—C21—C20	−178.2 (6)	C16—O6—C15—C18	177.4 (6)
C12—N1—C3—O3	−179.8 (6)	C9—C8—C5—N2	−177.9 (7)
C12—N1—C3—C4	0.1 (6)	C9—C8—C5—C4	0.4 (10)
C12—N1—C2—C13	−72.8 (7)	C22—S1—C1—C2	174.5 (5)
C12—N1—C2—C1	53.6 (8)	C19—O7—C18—C20	4.8 (10)
C12—C11—C4—C3	0.3 (7)	C19—O7—C18—C15	−176.4 (7)
C12—C11—C4—C5	−179.9 (6)		

### Hydrogen-bond geometry (Å, º)

**Table:**

*D*—H···*A*	*D*—H	H···*A*	*D*···*A*	*D*—H···*A*
N2—H2···O3	0.86	2.31	2.986 (7)	136
O8—H8*A*···O9	0.92	2.30	3.20 (3)	166
O9—H9*B*···O1	0.89	2.54	2.994 (17)	112
C8—H8···O4	0.93	2.30	2.894 (9)	121
C1—H1*A*···O5	0.97	2.45	3.068 (8)	121
C1—H1*A*···O5^i^	0.97	2.32	3.172 (8)	147
C1—H1*B*···O4^ii^	0.97	2.56	3.524 (9)	172
C14—H14···O5^i^	0.93	2.49	3.415 (8)	178
C19—H19*C*···O4^iii^	0.96	2.61	3.567 (10)	173
C20—H20···O2^ii^	0.93	2.46	3.370 (9)	166
C22—H22*C*···O3^iv^	0.96	2.44	3.088 (9)	124
C22—H22*C*···O9	0.96	2.61	3.36 (2)	136

Symmetry codes: (i) *y*, *x*, −*z*+1; (ii) *x*+1/2, −*y*+3/2, −*z*+3/4; (iii) −*y*+5/2, *x*+1/2, *z*+1/4; (iv) −*y*+3/2, *x*−1/2, *z*+1/4.
